# Perception, sentiments, and the level of awareness toward the dental implant among general population in Sulaimaniyah City, Iraq

**DOI:** 10.1186/s12903-024-03964-w

**Published:** 2024-02-20

**Authors:** Nzar Abdulqadr Muhammed Amin

**Affiliations:** Department of Oral and Maxillofacial Surgery, College of Dentistry, University of Sulaimani, Sulaimaniyah, 0046 Iraq

**Keywords:** Attitudes, Knowledge, Dental implant, Perception, Missing teeth

## Abstract

**Background:**

Edentulism is one of the most commonly encountered conditions affecting the oral cavity. Dental implants have emerged as a widely accepted treatment option prosthodontically. However, lack of public awareness and the cost of the treatment act as barriers to limit their applicability.

**Objectives:**

To investigate the perception, sentiments, and level of awareness toward dental implants among the general population in Sulaimaniyah City, Iraq.

**Materials and methods:**

A questionnaire-based survey was conducted on 1132 participants in Sulaimaniyah City, Iraq, from February 15, 2023, till August 15, 2023, to collect their sociodemographic data, knowledge and attitudes toward dental implants for their missing tooth/teeth.

**Results:**

The mean age of participants was 41.3 ± 14.6 years, and most (30.7%) belong to the age group 31–44 years. Also, most of them were males (63.6%), married (75.3%), educated (91.3%), from rural areas (82.3%), and had > 1 missing tooth (75%). Regarding the participants’ knowledge of dental implants, most had information (78.4%)/heard about it (83.3%), mainly from dentists (43.6%); however, least of them (21.5%) /their family members (43%) had replaced missing teeth by dental implants. Additionally, the participant’s attitudes toward the dental implant indicated that most of them were interested in replacing their missing teeth (88.8%) but did not do it due to financial reasons (87%) and considered replacement as a significant (92.5%) and safe process (79.2%). Also, most of them thought there was no substantial difference between artificial and natural tooth appearance/function (47.9%), preferred the delayed implant for dental implant (47.7%), and would like to do an implant due to problems in dental appearance, speech, or dental function (81.5%). Finally, significant/highly significant differences were seen between each participant’s factor (age, gender, marital status, education level, and residency) with most items of attitude/awareness toward dental implants.

**Conclusions:**

Most participants were aware of the knowledge and attitude of dental implants for replacing missing tooth/teeth, especially males, married ones, educated individuals, and those from urban areas; however, financial problems are the main obstacle. Additionally, the delayed implant is preferable for replacing missing tooth/teeth using dental implants among studied individuals.

**Supplementary Information:**

The online version contains supplementary material available at 10.1186/s12903-024-03964-w.

## Introduction

Tooth loss is a severe life event that impairs two essential functions, namely, eating and speaking, and has significant side effects on different quality of life [1]. A dental implant is an artificial root surgically inserted into the jawbone to support a single tooth replacement, fixed partial, complete denture or maxillofacial prosthesis [2]. It has become increasingly important as most patients have reported improved quality of life, assurance, and self-confidence, including psychological benefits and conservation of the tooth structure adjacent to the teeth to be replaced [3].

Due to dental implants’ high success rates and predictability, their clinical implication is rapidly increasing [1]. It was reported that the level of awareness of dental implant treatment was 23.24% in 2015 [4], while a study in the United States of America (USA) reported that dental implant prevalence projected to 2026 ranged from 5.7% in the most conservative scenario to 23% in the least [5].

There is no need to prepare the adjacent teeth for abutments if one is planning a three-unit bridge. Therefore, it is the most conservative tooth structure. In the case of dental implants, the abutments are used to join the bridge or other tooth replacements to the fixture inserted in the jawbone [6, 7].

Implant treatment has become the focus of the patient’s interest; hence, for dentists, it is vital to assess their level of knowledge about dental implants and whether their perception of dental implants reflects reality to guide patients who do not have the education or background knowledge to make an informed decision. The attitude towards tooth loss is changing. Adults have greater expectations of their dental health than in the past [8]. Now a day, aesthetic dentistry is growing. People are generally very concerned about their beauty and will follow the advanced technology in the medical and dental field. Dental implants play a vital role; thus, it is essential to know about the local population’s awareness, attitude, and knowledge about dental implants [9]. In this regard, Gbadebo et al. (2014) reported a low awareness level about dental implants as a tooth replacement option in Nigeria. However, most of their study participants knew missing teeth could be replaced [10]. Also, Padhye et al. (2019) stated that the Indian metropolitan population had heard about dental implants as a treatment option but considered them expensive. Improved doctor–patient communication would be needed to avoid misconceptions regarding implant therapy and its cost [11].

Although the exact number of dental implant treatments in Iraq, primarily the Kurdistan region, has yet to be discovered, evidence suggests that the demand for implant therapy is increasing, similar to other countries worldwide. Thus, there is a need to raise awareness about their potential complications. Professionals in the dental and medical fields must take responsibility for educating the public, and future professionals in these fields will play a vital role in this effort [12]. Therefore, a survey was conducted to determine the perception, sentiments, and level of awareness of implant treatment among the general population in Sulaimaniyah City, Iraq.

## Patients and methods

### Study design and setting

This cross-sectional, prospective, observational-based survey study was conducted randomly on 1132 participants in different locations/areas of Sulaimaniyah City, Iraq, from February 15, 2023, to August 15, 2023, using a simple random sampling technique.

### Questionnaire

Following Kuppuswamy’s classification [13, 14], a self-administered questionnaire was prepared and validated by five professionals in the field of dental implants and then used. The questionnaire was comprised of three parts. The first part was used to collect participant’s sociodemographic data, including age, gender, marital status, education level, and residency (Supplementary 1). The 2nd part comprised data collection on participants’ knowledge/awareness about dental implants (5 items), and 3rd part included participants’ data on attitudes toward dental implants (7 items). The questionnaire was initially prepared in English and translated into Kurdish (mother tongue) to facilitate completion and better understanding of the questions by the participants.

### Study area

Generally, most locations in Sulaimaniyah City were visited for data collection, including private clinics, government health centres, hospitals, evening schools, universities, institutes, and general libraries.

### Inclusion criteria

Participants aged < 18 to 86 years old with/without missing teeth, regardless of gender, ethnicity or nationality, were included in this study.

### Exclusion criteria

Participants on anti-coagulants, chemotherapy and radiotherapy were omitted, together with those who had psychological disturbances, such as manic, change in mood and disabled patients. Also, people with mental retardation, Down syndrome or any other psychiatric upset were excluded.

### Data analysis

Collected data was analyzed using Statistical Package for Social Science (SPSS, Version 27). The Chi-square test was used to determine the association between variables and different items of attitude/knowledge of dental implants among studied populations. A *p*-value of ≤ 0.05 was considered a significant difference, *p* ≤ 0.001 was set as a highly significant difference, and Cronbach’s Alpha score was 0.739.

## Results

The present study evaluated the participants’ knowledge, perception, attitude, and awareness toward dental implants as the best choice for missing teeth/teeth replacement among various treatment modalities. The mean age of participants was 41.3 ± 14.6 years, and most (30.3%) belonged to the age group 31–44 years. Also, most of them were males (63.6%), married (75.3%), educated (91.3%), and from rural areas (82.3%) (Table 1).

**Table 1:** Sociodemographic characteristics of studied participants

Sociodemographic characteristic	Frequency	Percentage
**Age (Years)**		
< 18	22	2.0
18–30	314	28.0
31–44	344	30.3
45–60	310	27.2
> 60	142	12.5
**Gender**		
Male	720	63.6
Female	412	36.4
**Marital status**		
Single	227	20.1
Married	853	75.3
Widow	49	4.3
Divorce	3.0	0.3
**Education level**		
Illiterate	98	8.7
Primary	144	12.7
Intermediate	132	11.7
Secondary	271	23.9
University	381	33.7
Higher education	106	9.3
**Residency**		
Rural	932	82.3
Urban	200	17.7
**Total**	**1132**	**100**

**Fig. 1:**
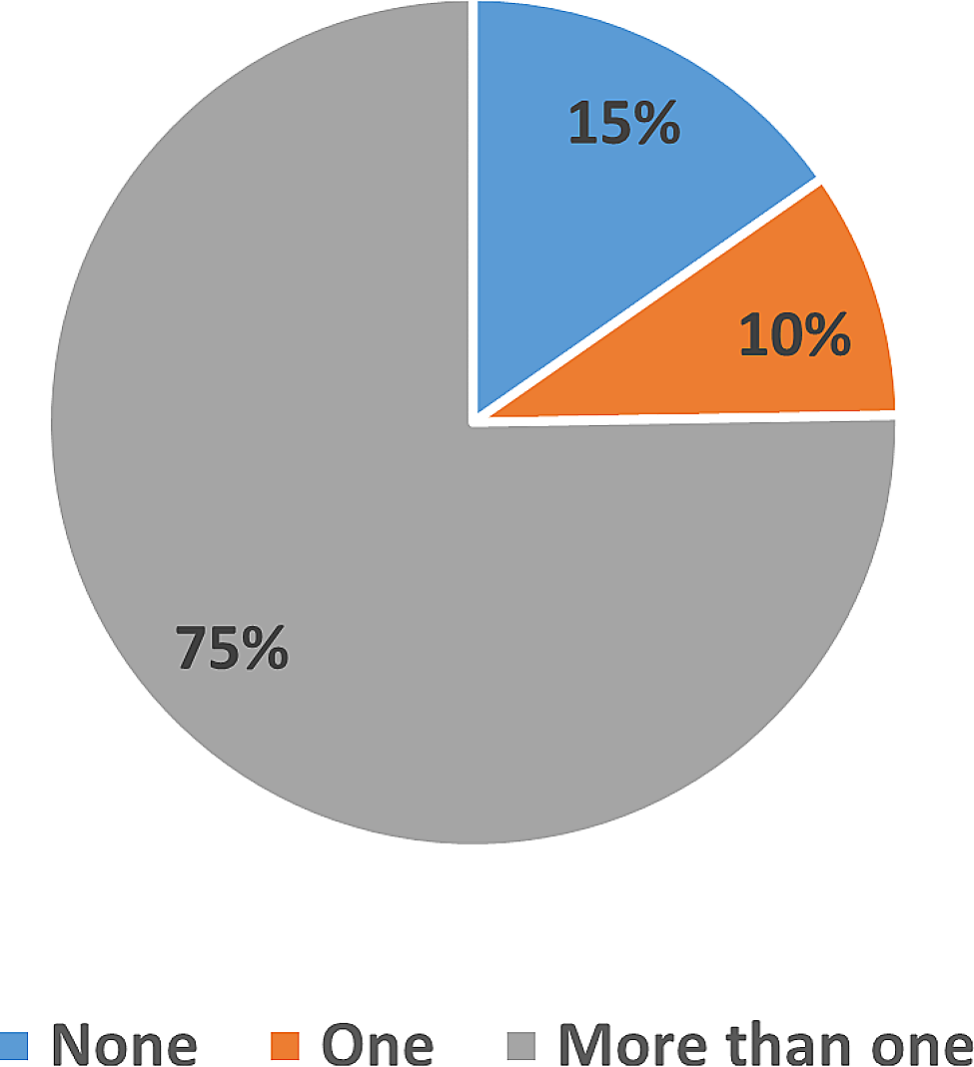
The prevalence of missing tooth/teeth in studied participants

Regarding the number of missing teeth, most participants had > one missing tooth (75%), least had one missing tooth (10%), and the rest had not (15%) (Fig. 1). When participants were enquired about knowledge of dental implants, most had information about it (78.4%) and heard about it (83.3%), especially from dentists (43.6%), while least of them (21.5%) and their family members (43%) reported to had replacement of their missing teeth using dental implants (Table 2).

**Table 2:** Participants knowledge about dental implant

Variable	Frequency	Percentage
**Have information about dental implant**		
Yes	887	78.4
No	245	21.6
**I heard about this treatment**		
Yes	943	83.3
No	189	16.7
**Source of information**		
Newspaper	5.0	0.4
Magazine	2.0	0.2
TV/Radio	22	1.9
Internet	35	3.1
Social media	159	14.0
Family	32	2.8
Society	121	10.7
School	7	0.6
University	59	5.2
Dentist	493	43.6
Other	12	1.1
Didn’t heard about it	185	16.3
**Undergone dental implant**		
Yes	243	21.5
No	889	78.5
**Family members have undergone dental implant**		
Yes	487	43
No	645	57
**Total**	**1132**	**100**

Moreover, regarding the participants’ attitude toward the dental implant, most of them were interested in replacing their missing teeth, but most did not replace their missing tooth/teeth due to financial reasons; most considered replacement of their missing teeth to be essential and safe (88.8%, 87%, 92.5%, and 79.2%, respectively). Also, most of them thought there was no substantial difference between artificial and natural teeth in appearance/function (47.9%), preferred the delayed implant for dental implant (47.7%), and would like to do an implant due to having problems in dental appearance, speech, or dental function (81.5%) (Table 3).

**Table 3:** Participants attitude toward dental implant

Variable	Frequency	Percentage
**Are you ready to replace lost tooth/teeth with an implant?**		
Yes	1005	88.8
No	41	3.6
I don’t know	86	7.6
**Reasons for not replacing the missing tooth/teeth**		
Financial reason	985	87
I did not feel the need	29	2.6
No time	61	5.4
Did not know	57	5.0
**The reason behind planning for dental implant**		
Appearance/Aesthetic	115	10.3
Speech	10	0.9
Function	82	7.3
More than one of them	910	81.5
**Do you think that dental implant is the best way for rehabilitation**		
Yes	1047	92.5
No	24	2.1
I don’t know	61	5.4
**Do you think that dental implant is a safe procedure**		
Yes	897	79.2
No	24	2.1
I don’t know	211	18.7
**Do you think artificial teeth are equivalent to natural teeth in appearance and function**		
Yes	542	47.9
No	451	39.8
I don’t know	139	12.3
**Which method of dental implant do you prefer**		
Conventional	540	47.7
Immediate	403	35.6
I don’t know	199	17.7
**Total**	**1132**	**100**

On correlating various factors related to the participants’ awareness about the importance of replacing their missing tooth/teeth with dental implants, it was observed that almost each age group considered a replacement of missing teeth necessary. Still, the awareness about replacing missing tooth/teeth with dental implants was minimal in the elderly participants (0.8%), mainly due to financial reasons (97.7%). However, they had more than a reason for replacement (86.2%). On the other hand, most participants in the age group 31–44 showed the highest rate for safety replacement procedures (95%) and high-quality artificial teeth (54.1%) and liked the delayed implant (85%). There was a highly significant difference (*p* < 0.001) between various items for all age groups, except for safety, which was substantial (*p* = 0.03) (Supplementary 2).

Regarding the correlations between the participants’ attitude and their gender, as indicated in Supplementary 3, a significant difference (*p* < 0.001) was seen for all items and gender, except for the equivalence of artificial teeth to natural teeth (*p* = 0.39). More often, males had financial support for replacing the missing tooth/teeth (91.5%), also had more than a reason for replacement (84.4%), and thought dental implant is a safe process (83.8%), especially when using delayed implant (49%).

Regarding the correlations between the participants’ attitude and their level of education, a significant difference (*p* < 0.001) was seen for all items regarding various education levels. Illiterate or primary school graduates had more financial problems to replace their missing tooth/teeth (95.2%), while most participants of University graduate/higher education level considered the dental implant as a safe process (90%), had more than one reason for dental implant (82.2%), thought that artificial tooth/teeth were equivalent to natural one (56.1%). Also, they preferred the delayed implant for replacement (55.6%) (Supplementary 4).

Concerning participants’ attitudes toward the dental implant and marital status, a significant difference (*p* < 0.001) was seen for all items. Widow and divorced participants had more financial issues not replacing the missing tooth/teeth (94.4%), while more married individuals planned to do dental implant for more than one cause (84.5%), considered the process as a safe method (83.3%), thought that artificial tooth/teeth were equivalent to natural one (50.6%). Also, they preferred the delayed implant for replacement (49.4%) (Supplementary 5).

Finally, most participants from urban areas (96%) desired to replace their missing tooth/teeth, but most could not because of financial reasons (95.5%) compared to those living in rural areas. Simultaneously, most participants from rural areas had more than one missing tooth (83%); they considered the process as a safe procedure (82.7%), especially when using a delayed implant (50.3%). A highly significant difference (*p* ≤ 0.001) was seen for most items (Supplementary 6).

## Discussion

Various treatment options are available for treating edentulism; dental implantation is the best choice [15]. A dental implant is a newer, near-natural tooth replacement option available to replace missing tooth/teeth. Their use has become progressively important as most patients with dental implants have reported improved quality of life [3]. Various levels of awareness regarding dental implants among populations have been discussed in many studies worldwide, raising valid concerns about the insufficient education and attitude toward dental implants at most levels [6, 16]. Thus, for the first time in Iraq, we planned to determine the correlations between participants’ factors and awareness/attitude/knowledge towards dental implants. Most of our participants (30.7%) were 31–44 years old, with a mean age of 41.3 ± 14.6 years. This might be due to more awareness and perception of oral health and dental implant treatment among young people. This finding does not agree with another study that found the mean age of participants to be 61 years old [17]. Also, most of the participants were males (63.6%), married (75.3%), educated (91.3%), from rural areas (82.3%), and had > 1 missing tooth (75%). This might be related to the fact that males and married people primarily work outside our country. Male predominance is not parallel to another study on awareness about replacing missing tooth/teeth [16, 18, 19]. At the same time, it agreed with another study in Saudi Arabia that found more male (69.44%) participants [20].

Also, most participants had information (78.4%)/heard about dental implants (83.3%), mainly from dentists (43.6%); however, least of them (21.5%)/their family members (43%) had replaced missing teeth/teeth. In this regard, Yao et al. 2017) mentioned that the primary information source about implant therapy was the dentist/hygienist (42%), and most participants (62.8%) were generally informed about implants. Still, only 17.7% felt confident with the information they had [21]. Additionally, another study stated that most participants (55.56%) were moderately knowledgeable, whereas 37.5% had good knowledge about dental implant procedures [20]. On the contrary, a study in India found that most people (73.8%) were unaware of dental implants [22].

Additionally, the participants’ attitudes toward dental implants indicated that most of them were interested in replacing their missing teeth (88.8%) but did not do it due to financial reasons (87%) and considered replacement as a safe process (79.2%). Also, most of them thought there was no substantial difference between artificial and natural tooth appearance/function (47.9%), preferred the delayed implant for dental implant (47.7%), and would like to do an implant due to problems in dental appearance, speech, or dental function (81.5%). Similarly, Yao et al. 2017 stated that > 30% of participants appeared to maintain dangerous misperceptions about dental implants, and 52.7% considered dental implants functional as natural teeth [21]. When participants were asked about dental implants being the preferred choice for replacing missing dentition, most answered yes (92.5%), believing dental implantation was the best choice. These results were similar to a study conducted in Nigeria [10]. On the other hand, a systematic review observed that most patients had high expectations, with function followed by aesthetics being the most critical expected improvements, especially for women [23]. Additionally, a study in Japan stated that the most common perceptions of implant therapy were expensive (45%), advanced (38%), and scary (25%), while patients’ implant knowledge came primarily from magazines or books. At the same time, professional dental societies/associations were the most minor sought-after source of information [24]. Whereas the knowledge related to dental implants could have been more satisfactory, only 4.5% showed good understanding. The willingness to replace the current prosthesis with a dental implant was 49.6% [25].

Moreover, in this study, the awareness about replacing missing tooth/teeth with dental implants was minimal in the elderly participants (0.8%), mainly due to financial reasons (97.7%). However, they had more than a reason for replacement (86.2%). On the other hand, most participants in the age group 31–44 showed the highest rate for safety replacement procedures (95%) and high-quality artificial teeth (54.1%) and liked the delayed implant (85%). Another study stated that younger subjects (< 45 years) and those with higher education levels (bachelor and postgraduate) tended to present more realistic perceptions and lower outcome expectations [21].

Furthermore, regarding gender, in this study, most males had financial support for replacing the missing tooth/teeth (91.5%), also had more than a reason for replacement (84.4%), and thought dental implant is a safe process (83.8%), especially when using the delayed implant (49%). Thus, it was encouraging that males had almost more knowledge, perception and awareness about replacing missing tooth/teeth with dental implants than females. The reason could be unequal opportunities and literacy rates among males and females. Also, a significant difference (*p* < 0.001) was seen for all items of attitude and participants’ gender, except for the equivalence of artificial teeth to natural teeth (*p* = 0.39). This outcome is similar to another study that found no significant association between the knowledge and gender of the participants [20].

Consequently, a significant correlation (*p* < 0.001) was seen for all items of participants’ attitudes and their level of education. Illiterate/primary school graduates had more financial issues (95.2%) with dental implants. Most University graduates and people with higher education levels considered dental implants as a safe process (90%), had more than one reason for dental implants (82.2%), and regarded artificial tooth/teeth to be equivalent to natural ones (56.1%). Also, this group predominantly preferred the delayed implant for dental replacement (55.6%). Thus, education significantly influences patient awareness/attitude about dental implants.

Also, a significant difference (*p* < 0.001) was seen for all participants’ attitudes toward dental implants and marital status. Widow and divorced participants had more financial issues not replacing the missing tooth/teeth (94.4%), while more married individuals planned to do dental implant for more than one cause (84.5%) and considered the process as a safe method (83.3%), thought that artificial tooth/teeth were equivalent to natural one (50.6%), and also they preferred the delayed implant for replacement (49.4%).

Ultimately, most participants from urban areas (96%) anticipated having dental implants, but most could not do it because of financial reasons (95.5%). In comparison, most participants from rural areas had more than one missing tooth (83%) and considered the process as a safe procedure (82.7%), especially when using the delayed implant (50.3%). A highly significant difference (*p* ≤ 0.001) was seen for most awareness items towards dental implants and residency.

Significant/highly significant differences were seen between each participant’s factor (age, gender, marital status, education level, and residency) with most items of attitude/awareness toward dental implants. These findings agreed with another study that showed a significant association between the volunteers’ occupation, educational levels, and age group toward knowledge of dental implants [22]. Another study found that the awareness of dental implants was significantly (*p* < 0.05) affected by the participants’ age, residency, and level of education [26]. Thus, educating people about dental implants was encouraging as we received an overwhelmingly positive response from patients who would like to know more about them. Similar results were seen in a study conducted in India [27].

## Conclusions

Within the limitations of this study, it was found that the study participants were eager to choose dental implants but were restricted due to financial issues. Also, there is a significant difference between the knowledge and information about the importance of replacing missing teeth and the perception of recent and new replacement options.

### Limitations and recommendations

Although dental implant procedures in dentistry have shown promising results, there is a need to spread awareness and overcome the associated barriers that limit the applicability of dental implants. Dental health professionals and the government should make a combined effort with sites of reclaims like TV programming to overcome the obstacles, such as bone quality and quantity, healing time, infections at the implant site, surgical risks, complications such as implant failure, nerve damage, and sinus problems, post-operative oral care, and health factors, including certain medical conditions and medications that can affect the success of dental implants.

### Electronic supplementary material

Below is the link to the electronic supplementary material.

Supplementary Material 1

Supplementary Material 2

## Data Availability

The datasets used and analyzed during the current study are available from the corresponding author upon reasonable request.
