# Supplementary material for: Perception, sentiments, and the level of awareness toward the dental implant among general population in Sulaimaniyah City, Iraq
Source: BMC Oral Health. 2024 Feb 20;24:255. doi: 10.1186/s12903-024-03964-w (PMC10877875; doi:10.1186/s12903-024-03964-w)
Supplement: Supplementary file 1 — Supplementary Material 1 [file 12903_2024_3964_MOESM1_ESM.docx]

**Supplementary 1A.** Questions on sociodemographic characteristics of studied participants.

| **Sociodemographic characteristic** | **Frequency** | **Percentage** |
| --- | --- | --- |
| **Age (Years)** | | |
| <18 |  |  |
| 18 - 30 |  |  |
| 31 - 44 |  |  |
| 45 - 60 |  |  |
| >60 |  |  |
| **Gender** | | |
| Male |  |  |
| Female |  |  |
| **Marital status** | | |
| Single |  |  |
| Married |  |  |
| Widow |  |  |
| Divorce |  |  |
| **Education level** | | |
| Illiterate |  |  |
| Primary |  |  |
| Intermediate |  |  |
| Secondary |  |  |
| University |  |  |
| Higher education |  |  |
| **Residency** | | |
| Rural |  |  |
| Urban |  |  |
| **Total** |  |  |

**Supplementary 1B.** Questions on participants’ knowledge about dental implants.

| **Variable** | **Frequency** | **Percentage** |
| --- | --- | --- |
| **Have information about dental implant** | | |
| Yes |  |  |
| No |  |  |
| **I heard about this treatment** | | |
| Yes |  |  |
| No |  |  |
| **Source of information** | | |
| Newspaper |  |  |
| Magazine |  |  |
| TV/Radio |  |  |
| Internet |  |  |
| Social media |  |  |
| Family |  |  |
| Society |  |  |
| School |  |  |
| University |  |  |
| Dentist |  |  |
| Other |  |  |
| I have yet to hear about it. |  |  |
| **Undergone dental implant** | | |
| Yes |  |  |
| No |  |  |
| **Family members have undergone dental implant** | | |
| Yes |  |  |
| No |  |  |
| **Total** |  |  |

**Supplementary 1C.** Questions on participants’ attitudes toward dental implants.

| **Variable** | **Frequency** | **Percentage** |
| --- | --- | --- |
| **Are you ready to replace lost teeth with an implant** | | |
| Yes |  |  |
| No |  |  |
| I don't know. |  |  |
| **Reasons for not replacing the missing tooth/teeth** | | |
| Financial reason |  |  |
| I did not feel the need. |  |  |
| No time |  |  |
| Did not know |  |  |
| **The reason behind planning for dental implant** | |  |
| Appearance/Aesthetic |  |  |
| Speech |  |  |
| Function |  |  |
| More than one of them |  |  |
| **Do you think that dental implant is the best way for rehabilitation** | | |
| Yes |  |  |
| No |  |  |
| I don't know. |  |  |
| **Do you think that a dental implant is a safe procedure?** | |  |
| Yes |  |  |
| No |  |  |
| I don't know. |  |  |
| **Do you think artificial teeth are equivalent to natural teeth in appearance and function** | | |
| Yes |  |  |
| No |  |  |
| I don't know. |  |  |
| **Which method of dental implant do you prefer** | | |
| Conventional |  |  |
| Immediate |  |  |
| I don't know. |  |  |
| **Total** |  |  |
