# Supplementary material for: Perception, sentiments, and the level of awareness toward the dental implant among general population in Sulaimaniyah City, Iraq
Source: BMC Oral Health. 2024 Feb 20;24:255. doi: 10.1186/s12903-024-03964-w (PMC10877875; doi:10.1186/s12903-024-03964-w)
Supplement: Supplementary file 2 — Supplementary Material 2 [file 12903_2024_3964_MOESM2_ESM.docx]

**Supplementary 2.** Comparing the participants' attitudes with age.

| **Variable** | **Age (Years)** | | | | | | | | | | | | **p-value** |
| --- | --- | --- | --- | --- | --- | --- | --- | --- | --- | --- | --- | --- | --- |
|  | **<18** | | **18 - 30** | | **31 - 44** | | **45 - 60** | | **>60** | | **Total** | |  |
|  | **Number, %** | | | | | | | | | | | |  |
| **Are you ready to replace your lost tooth/teeth?** | | | | | | | | | | | | |  |
| Yes | 33 | 78.5 | 250 | 80.9 | 307 | 89.8 | 285 | 92.8 | 129 | 97.7 | 1004 | 88.5 | <0.001** |
| No | 3.0 | 7.1 | 20 | 6.5 | 12 | 3.5 | 5.0 | 1.6 | 1.0 | 0.8 | 41 | 3.7 |  |
| I don’t know | 4.0 | 9.5 | 12 | 3.9 | 9.0 | 2.6 | 10 | 3.3 | 1.0 | 0.8 | 36 | 3.2 |  |
| I didn’t think about it | 2.0 | 4.9 | 27 | 8.7 | 14 | 4.1 | 7.0 | 2.3 | 1.0 | 0.8 | 51 | 4.6 |  |
| **Reasons for not replacing the missing tooth/teeth** | | | | | | | | | | | | | |
| Financial reason | 31 | 67.4 | 253 | 82.7 | 306 | 89.7 | 282 | 91.6 | 128 | 97.7 | 1000 | 88.1 | <0.001** |
| I did not feel the need | 2.0 | 4.3 | 14 | 4.6 | 7.0 | 2.1 | 6.0 | 1.9 | 0.0 | 0.0 | 29 | 2.6 |  |
| No time | 1.0 | 2.2 | 13 | 4.2 | 19 | 5.6 | 11 | 3.6 | 2.0 | 1.5 | 46 | 4.2 |  |
| Did not know | 12 | 26.1 | 26 | 8.5 | 9 | 2.6 | 9.0 | 2.9 | 1.0 | 0.8 | 57 | 5.1 |  |
| **Reason for planning for dental implant** | | | | | | | | | | | | | |
| Appearance/Aesthetic | 10 | 21.7 | 51 | 16.6 | 31 | 9.1 | 20 | 6.5 | 3.0 | 2.3 | 115 | 10.1 | <0.001** |
| Speech | 0.0 | 0.0 | 4.0 | 1.3 | 5.0 | 1.5 | 0.0 | 0.0 | 1.0 | 0.8 | 10 | 0.9 |  |
| Function | 2.0 | 4.3 | 14 | 4.5 | 18 | 5.3 | 34 | 11.1 | 14 | 10.8 | 82 | 7.4 |  |
| More than one of them | 34 | 73.9 | 239 | 77.6 | 288 | 84.2 | 252 | 82.4 | 112 | 86.2 | 925 | 81.6 |  |
| **Is a dental implant a safe procedure?** | | | | | | | | | | | | |  |
| Yes | 17 | 41.5 | 290 | 92.9 | 325 | 95 | 290 | 94.5 | 117 | 90 | 1039 | 92 | 0.03* |
| No | 0.0 | 0.0 | 6.0 | 1.9 | 1.0 | 0.3 | 2.0 | 0.7 | 0.0 | 0.0 | 9.0 | 0.8 |  |
| I don't know | 24 | 58.5 | 16 | 5.1 | 16 | 4.7 | 15 | 4.9 | 13 | 10 | 84 | 7.2 |  |
| **Do you think artificial teeth are equivalent to natural teeth in appearance and function?** | | | | | | | | | | | | | |
| Yes | 17 | 40.5 | 168 | 53.7 | 184 | 54.1 | 146 | 47.1 | 36 | 28.3 | 551 | 48.7 | <0.001** |
| No | 12 | 28.5 | 115 | 36.7 | 122 | 35.9 | 125 | 40.3 | 69 | 54.3 | 443 | 39.1 |  |
| I don't know | 13 | 31 | 30 | 9.6 | 34 | 10 | 39 | 12.6 | 22 | 17.3 | 138 | 12.2 |  |
| **The preferred method for dental implant** | | | | | | | | | | | | | |
| Conventional | 11 | 52.4 | 260 | 83.3 | 308 | 85 | 254 | 82.7 | 78 | 60 | 911 | 80.1 | <0.001** |
| Immediate | 1.0 | 4.8 | 6.0 | 1.9 | 6.0 | 1.8 | 1.0 | 0.3 | 0.0 | 0.0 | 14 | 1.3 |  |
| I don't know. | 9.0 | 42.9 | 46 | 14.7 | 48 | 14 | 52 | 16.9 | 52 | 40 | 207 | 18.6 |  |
| **Total** | 21 | 100 | 312 | 100 | 362 | 100 | 307 | 100 | 130 | 100 | 1132 | 100 |  |

*: Significant difference; **: Highly significant difference using Chi-square test

**Supplementary 3.** Comparing the participants’ attitudes with gender.

| **Variable** | **Male** | | **Female** | | **Total** | | **p-value** |
| --- | --- | --- | --- | --- | --- | --- | --- |
|  | **Number, %** | | | | | |  |
| **Reasons for not replacing the missing tooth/teeth** | | | | | | |  |
| Financial reason | 659a | 91.5 | 326b | 79.1 | 985 | 87.0 | <0.001* |
| I did not feel the need | 23a | 3.2 | 36b | 8.7 | 59 | 5.2 |  |
| No time | 25a | 3.5 | 21a | 5.1 | 46 | 4.1 |  |
| Did not know | 13a | 1.8 | 29b | 7.1 | 42 | 3.7 |  |
| **Reason for planning for dental implant** | | | | | | | |
| Appearance/Aesthetic | 61a | 8.5 | 69b | 16.7 | 130 | 11.5 | <0.001* |
| Speech | 3.0a | 0.4 | 7.0b | 1.7 | 10 | 0.9 |  |
| Function | 48a | 6.7 | 34a | 8.3 | 82 | 7.2 |  |
| More than one of them | 608a | 84.4 | 302b | 73.3 | 910 | 80.4 |  |
| **Is a dental implant a safe procedure?** | | | | | | | |
| Yes | 603a | 83.8 | 304b | 73.8 | 907 | 80.1 | <0.001* |
| No | 7.0a | 1.0 | 7.0a | 1.7 | 14 | 1.3 |  |
| I don't know | 110a | 15.2 | 101b | 24.5 | 211 | 18.6 |  |
| **Do you think artificial teeth are equivalent to natural teeth in appearance and function?** | | | | | | | |
| Yes | 358a | 49.8 | 195a | 47.3 | 553 | 48.9 | 0.39 |
| No | 279a | 38.7 | 172a | 41.8 | 451 | 39.8 |  |
| I don't know | 83a | 11.5 | 45a | 10.9 | 128 | 11.3 |  |
| **The preferred method for dental implant** | | | | | | | |
| Conventional | 353a | 49 | 178b | 43.2 | 531 | 46.9 | <0.001* |
| Immediate | 265a | 36.8 | 137a | 33.3 | 402 | 35.5 |  |
| I don't know | 102a | 14.2 | 97b | 23.5 | 199 | 17.6 |  |
| **Total** | 720 | 100 | 412 | 100 | 1132 | 100 |  |

*: Highly significant difference using the Chi-square test

Using the Bonferroni method, a similar subscript letter denotes a subset of gender categories whose column proportions do not differ significantly from each other at the p=0.05

**Supplementary 4.** Comparing the participants' attitudes with educational levels.

| **Attitude** | **Illiterate/Primary** | | **Intermediate/Secondary** | | **University/ Higher** | | **Total** | | **p-value** |
| --- | --- | --- | --- | --- | --- | --- | --- | --- | --- |
|  | **Number, %** | | | | | | | |  |
| **Reasons for not replacing the missing tooth/teeth** | | | | | | | | |  |
| Financial reason | 231a | 95.2 | 365a,b | 90.6 | 421b | 86.4 | 1015 | 89.7 | 0.004* |
| I did not feel the need | 2.0a | 0.7 | 11a | 2.7 | 16a | 3.3 | 29 | 2.6 |  |
| No time | 5.0a | 2.0 | 9.0a | 2.2 | 32b | 6.6 | 46 | 4.0 |  |
| Did not know | 6.0a | 2.1 | 18a | 4.5 | 18a | 3.7 | 42 | 3.7 |  |
| **Reason for planning for dental implant** | | | | | | | | | |
| Appearance/Aesthetic | 18a | 7.4 | 41a | 10.2 | 56a | 11.5 | 115 | 10 | <0.001* |
| Speech | 1.0a | 0.4 | 3.0a | 0.7 | 6.0a | 1.2 | 10 | 0.9 |  |
| Function | 34a | 14.1 | 23b | 5.7 | 25b | 5.1 | 82 | 7.4 |  |
| More than one of them | 189a | 78.1 | 336a | 83.4 | 400a | 82.2 | 925 | 81.7 |  |
| **Is a dental implant a safe procedure?** | | | | | | | | | |
| Yes | 137a | 56.6 | 332b | 82.4 | 439c | 90 | 908 | 80.2 | <0.001* |
| No | 1.0a | 0.4 | 6.0a | 1.5 | 7.0a | 1.5 | 14 | 1.2 |  |
| I don't know | 104a | 43.5 | 65b | 16.1 | 41c | 8.5 | 210 | 18.6 |  |
| **Do you think artificial teeth are equivalent to natural teeth in appearance and function?** | | | | | | | | | |
| Yes | 68a | 28 | 200b | 49.6 | 273b | 56.1 | 541 | 47.7 | <0.001* |
| No | 110a | 45.5 | 166a | 41.2 | 188a | 38.6 | 464 | 41 |  |
| I don't know | 64a | 26.5 | 37b | 9.2 | 26b | 5.3 | 127 | 11.3 |  |
| **The preferred method for dental implant** | | | | | | | | | |
| Conventional | 59a | 24.4 | 197b | 48.8 | 271b | 55.6 | 527 | 46 | <0.001* |
| Immediate | 121a | 50 | 145b | 36 | 143b | 29.4 | 418 | 37 |  |
| I don't know | 62a | 25.6 | 61b | 15.1 | 73b | 15 | 196 | 17 |  |
| **Total** | 242 | 100 | 403 | 100 | 487 | 100 | 1132 | 100 |  |

*: Highly significant difference using the Chi-square test

Using the Bonferroni method, a similar subscript letter denotes a subset of educational categories whose column proportions do not differ significantly from each other at p=0.05

**Supplementary 5.** Comparing the participants' attitudes with marital status.

| **Variable** | **Single** | | **Married** | | **Widow/divorce** | | **Total** | | **p-value** |
| --- | --- | --- | --- | --- | --- | --- | --- | --- | --- |
|  | **Number, %** | | | | | | | |  |
| **Reasons for not replacing the missing tooth/teeth** | | | | | | | | | |
| Financial reason | 178a | 76.7 | 788b | 92.3 | 50b | 94.4 | 1,016 | 89.7 | <0.001* |
| I did not feel the need | 15a | 7.1 | 14b | 1.7 | 0.0b | 0.0 | 29 | 2.5 |  |
| No time | 11a | 5.2 | 33a | 3.9 | 1.0a | 2.8 | 45 | 4.0 |  |
| Did not know | 23a | 11.0 | 18b | 2.1 | 1.0b | 2.8 | 42 | 3.8 |  |
| **If you have a plan to do a dental implant, for which reason you do it** | | | | | | | | | |
| Appearance/Aesthetic | 45a | 19.8 | 69b | 8.1 | 2.0b | 3.8 | 116 | 10.2 | <0.001* |
| Speech | 4.0a | 1.8 | 6.0a | 0.7 | 0.0a | 0.0 | 10 | 0.9 |  |
| Function | 14a | 6.2 | 57a | 6.7 | 10b | 19.3 | 81 | 7.2 |  |
| More than one of them | 164a | 72.2 | 721b | 84.5 | 40a | 76.9 | 925 | 81.7 |  |
| **Is a dental implant a safe procedure?** | | | | | | | | | |
| Yes | 171a | 75.3 | 711b | 83.3 | 12c | 23 | 894 | 78.9 | <0.001* |
| No | 5.0a | 2.2 | 9.0a | 1.1 | 0.0a | 0.0 | 14 | 1.2 |  |
| I don't know | 51a | 22.5 | 133b | 15.6 | 40c | 77 | 224 | 19.9 |  |
| **Do you think artificial teeth are equivalent to natural teeth in appearance and function?** | | | | | | | | | |
| Yes | 105a | 46.2 | 432a | 50.6 | 10b | 19.4 | 547 | 48.3 | <0.001* |
| No | 99a | 43.6 | 331a | 38.8 | 30a | 57.6 | 460 | 40.6 |  |
| I don't know | 23a | 10.2 | 90a | 10.6 | 12b | 23 | 125 | 11.1 |  |
| **If you want to do a dental implant, which method do you prefer?** | | | | | | | | | |
| Conventional | 99a | 43.6 | 421a | 49.4 | 4.0b | 7.7 | 524 | 46.2 | <0.001* |
| Immediate | 57a | 25.1 | 319b | 37.4 | 28b | 53.8 | 404 | 35.6 |  |
| I don't know | 71a | 31.3 | 113b | 13.2 | 20a | 38.5 | 204 | 18.2 |  |
| **Total** | 227a | 100 | 853 | 100 | 52 | 100 | 1132 | 100 |  |

*: Highly significant difference using the Chi-square test

Using the Bonferroni method, a similar subscript letter denotes a subset of marital categories whose column proportions do not differ significantly from each other at the p=0.05

**Supplementary 6.** Comparing the participants' attitudes with their residency.

| **Variable** | **Rural** | | **Urban** | | **Total** | | **p-value** |
| --- | --- | --- | --- | --- | --- | --- | --- |
|  | **Number, %** | | | | | |  |
| **Are you ready to replace your missing tooth/teeth?** | | | | | | |  |
| Yes | 814a | 87.3 | 192b | 96 | 1,006 | 88.9 | 0.002** |
| No | 38a | 4.0 | 2.0a | 1.0 | 40 | 3.5 |  |
| I don’t know | 30a | 3.3 | 5.0a | 2.5 | 35 | 3.1 |  |
| I didn’t think about it | 50a | 5.4 | 1.0a | 0.5 | 51 | 4.5 |  |
| **Reasons for not replacing the missing tooth/teeth** | | | | | | | |
| Financial reason | 851a | 91.3 | 191a | 95.5 | 1,042 | 92 | 0.24 |
| I did not feel the need | 25a | 2.7 | 4.0a | 2.0 | 29 | 2.7 |  |
| No time | 42a | 4.5 | 4.0a | 2.0 | 46 | 4.0 |  |
| I don’t know | 14a | 1.5 | 1.0a | 0.5 | 15 | 1.3 |  |
| **Reason for planning for dental implant** | | | | | | | |
| Appearance/ Aesthetic | 94a | 10 | 20a | 10 | 114 | 10.1 | 0.01* |
| Speech | 6.0a | 0.7 | 4.0a | 2.0 | 10 | 0.9 |  |
| Function | 58a | 6.3 | 24b | 12 | 82 | 7.2 |  |
| More than one of them | 774a | 83 | 152b | 76 | 926 | 81.8 |  |
| **Is a dental implant a safe procedure?** | | | | | | | |
| Yes | 773a | 82.7 | 137b | 68.0 | 910 | 80.1 | <0.001** |
| No | 13a | 1.4 | 1.0a | 0.5 | 14 | 1.3 |  |
| I don't know | 146a | 15.9 | 62a | 31.5 | 208 | 18.6 |  |
| **Do you think artificial teeth are equivalent to natural teeth in appearance and function?** | | | | | | | |
| Yes | 479a | 51.4 | 73b | 36.5 | 552 | 48.5 | 0.001** |
| No | 359a | 38.5 | 94b | 47 | 453 | 40.1 |  |
| I don't know | 94a | 10.1 | 33a | 16.5 | 127 | 11.4 |  |
| **The preferred method for dental implant** | | | | | | | |
| Conventional | 469a | 50.3 | 60 | 30 | 529 | 46.6 | <0.001** |
| Immediate | 310a | 33.2 | 96 | 48 | 406 | 36 |  |
| I don't know | 153a | 16.5 | 44 | 22 | 197 | 17.4 |  |
| **Total** | 932a | 100 | 200 | 100 | 1132 | 100 |  |

*: Significant difference; **: Highly significant difference using Fisher exact test

Using the Bonferroni method, a similar subscript letter denotes a subset of residential categories whose column proportions do not differ significantly from each other at the p=0.05
